# Harnessing Artificial Intelligence for Hypothesis Generation in Childhood Asthma: Insights from NHANES

**DOI:** 10.70322/jrbtm.2026.10003

**Published:** 2026-04-28

**Authors:** Jing Liu, Yueh-Ying Han, Xiangyu Ye, Franziska J. Rosser, Kristina M. Gaietto, Chongyue Zhao, Wei Chen, Juan C. Celedón

**Affiliations:** 1 Division of Pediatric Pulmonary Medicine, UPMC Children’s Hospital of Pittsburgh, University of Pittsburgh, Pittsburgh, PA 15224, USA; 2 Department of Biostatistics and Health Data Science, School of Public Health, University of Pittsburgh, Pittsburgh, PA 15261, USA

**Keywords:** Artificial intelligence, Asthma, Children, Risk factors

## Abstract

Although large language models (LLMs) have undergone substantial development, their applicability to epidemiological research has not been sufficiently examined. This study aims to develop and evaluate an LLM-based framework for hypothesis generation and testing, demonstrating its application in childhood asthma in the National Health and Nutrition Examination Survey (NHANES). Pilot study was conducted to explore factors associated with childhood asthma in the 2001–2020 NHANES cycles. A modular agent system was developed, including Database Query, Statistic, Paper Search, and Paper Download tools, along with two LLM models (Key Generator and Hypothesis Tester). Multivariable logistic regression was used to test for the association between each variable and current asthma, generating a tentative affirmative claim. The Key Generator module produced keywords for literature search, the Paper Search and Paper Download tools queried PubMed and retrieved relevant studies, and the Hypothesis Tester module synthesized evidence and determined the support for claims for each variable. Keywords and conclusions were reviewed by researchers and validated using multiple LLMs (ChatGPT, DeepSeek, and Gemini) to ensure consistency and robustness. 25,839 children with (*n* = 2928) and without (*n* = 22,911) current asthma, and 10,359 variables were included in the multivariable analysis, which yielded 100 variables associated with asthma. Of these, 21 were directly related to asthma (supporting published studies), 43 were indirectly related to asthma (based on background knowledge, though not explicitly discussed in the available publications), and 34 were unrelated to asthma. Two variables were excluded due to a lack of discriminative keywords. This study demonstrates the effectiveness of LLM-based models for generating and testing hypotheses about childhood asthma.

## 1. Introduction

Large language models (LLM) can understand human language and formulate responses interpretable by humans. LLMs perform well on a wide range of natural language processing tasks, including language understanding, text generation, sentiment analysis, and machine translation. Prominent examples of current LLM include OpenAI’s ChatGPT [1], Google DeepMind’s Gemini [2], Anthropic’s Claude [3], Meta’s LLaMA [4], and Mistral’s Mixtral [5]. LLM has been used by researchers in domain-specific scenarios. For example, a pilot study demonstrated the feasibility of generating clinical letters for patients with average reading ability using ChatGPT [6].

An important goal of epidemiologic research is to investigate protective or risk factors for diseases. Researchers typically start with a hypothesis that is tested using available data [7–13]. Formulating a proper hypothesis typically requires advanced knowledge, including synthesizing evidence, identifying research gaps, and conceptualizing the research question—a process that is time-consuming and demands a high level of expertise but may be facilitated by LLM.

LLM has potential uses in biomedical research but can generate false answers that may seem correct (“Artificial Hallucinations”) [14]. Such hallucinations are not permissible in research settings where accuracy and reliability are critical.

The root causes of artificial hallucinations stem from fundamental limitations of LLM, including a lack of access to up-to-date or proprietary data and limited reasoning capabilities, particularly in domains such as mathematical reasoning. Thus, LLMs often struggle to incorporate constantly updated knowledge or proprietary resources, such as specialized databases [15].

One approach to mitigating hallucinations is domain-specific training. ProtGPT2 has been trained to understand protein sequences, supporting protein design and engineering [16], while BioGPT, trained on PubMed abstracts, has demonstrated strong performance in text generation and information extraction tasks [17]. However, fine-tuning often reduces versatility, resulting in diminished performance on tasks outside the training domain and increasing the risk of model overfitting.

A more lightweight solution involves the use of engineered prompts. GeneGPT has demonstrated how in-context learning, combined with carefully crafted prompts, can enable LLM to interact with the National Center for Biotechnology Information (NCBI) Web APIs to answer genomics questions [18].

Strategies to mitigate hallucinations range from established techniques—such as prompt engineering, retrieval-augmented generation (RAG) [19,20], and chain-of-thought (CoT) [21] distillation—to developing novel architectures that combine algorithmic innovation with improved data quality. Researchers have also explored flexible, modular systems that integrate language models with symbolic tools (e.g., APIs), enhancing adaptability and reliability [15]. An example is PaperQA [22], an agent-based RAG framework specifically designed to process scientific literature at scale.

Building on these advances, we aimed to leverage LLM while mitigating the risk of hallucinations by integrating components of data query, statistical analysis, and evidence-based reasoning (RAG enhanced). To evaluate its effectiveness and credibility, we conducted a pilot study exploring factors potentially associated with asthma, the most common chronic respiratory disease of childhood, utilizing data from participants in the National Health and Nutrition Examination Survey (NHANES). While numerous studies have identified a wide range of factors linked to asthma (e.g., genetic and environmental), a comprehensive analysis of potential protective or risk factors could provide deeper insights into this disease.

## 2. Statement of Significance

1. Problem or Issue: Asthma is the most common chronic respiratory disease of childhood. The causes of childhood asthma remain insufficiently understood.
2. What is Already Known: Potential protective or risk factors for asthma have been identified through traditional epidemiologic studies. Identifying new asthma-associated factors in large databases can be time-consuming and demands high expertise, but may be facilitated by LLM modeling.
3. What this Paper Adds: Our results highlight the potential use of LLMs in helping conduct efficient clinical research in asthma and other childhood diseases.
4. Who would benefit from the new knowledge in this paper: Clinicians, epidemiologists, and public health practitioners interested in efficient mining of public databases to identify novel risk factors for common childhood diseases, including asthma.

## 3. Methods

### 3.1. Data Sources and Study Sample

NHANES, conducted by the National Center for Health Statistics of the Centers for Disease Control and Prevention, is a nationwide survey designed to evaluate the health and nutritional status of adults and children in the U.S. All data used in this analysis were freely available and obtained from the NHANES website (https://www.cdc.gov/nchs/nhanes/index.html, accessed on 14 April 2026). We included data for 10,359 distinct variables from 25,839 children (ages 6–17 years) who participated in the NHANES cycles from 2001 to 2020. Current asthma was defined by a positive answer to both of the following questions: “Has a doctor or other health professional ever told you that you have asthma?” and “Do you still have asthma?”. Participants who answered no to both questions were selected as controls.

### 3.2. Framework and Workflow

**Figure 1.**
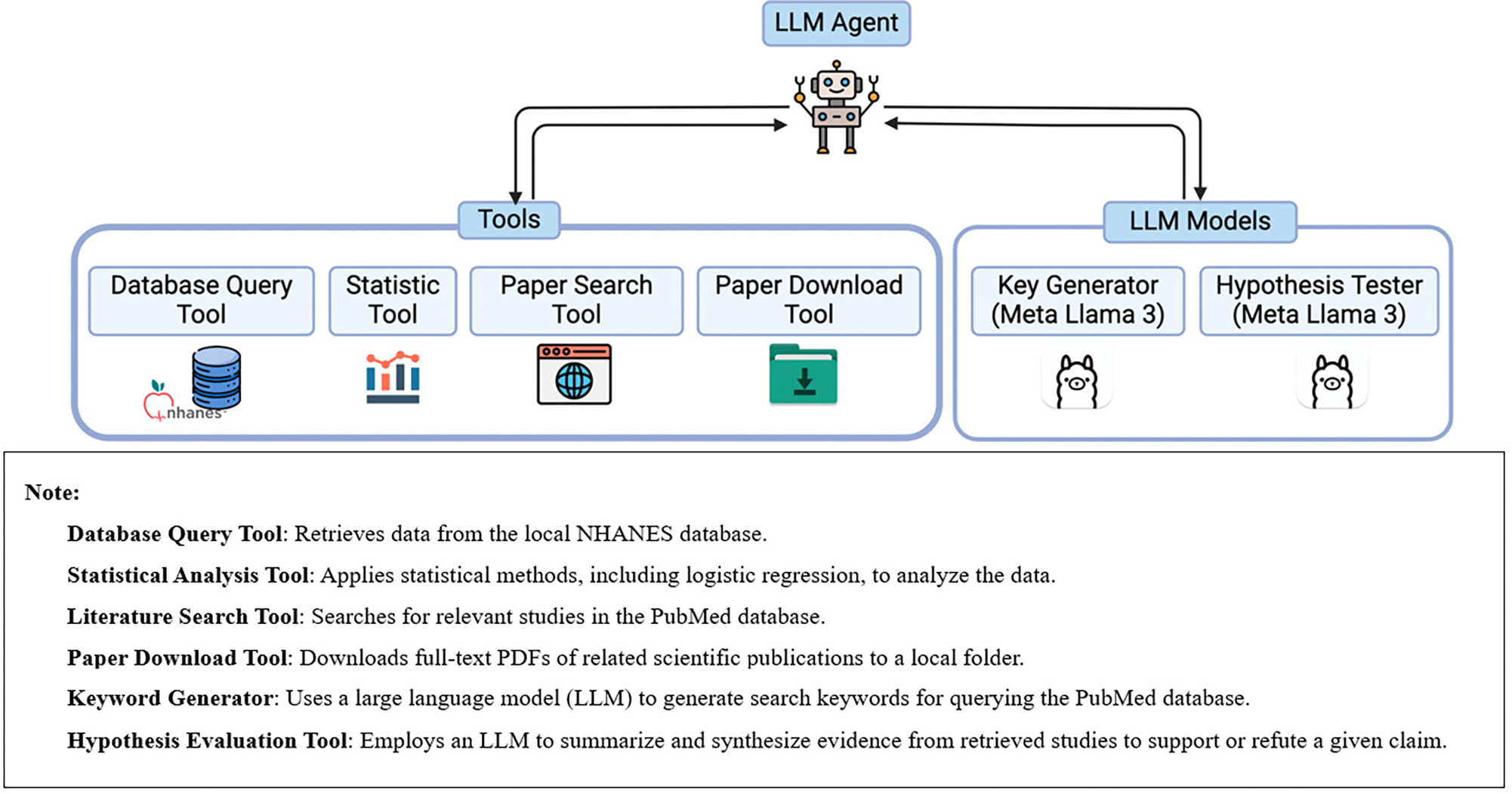
AI-Aided Hypothesis Generating & Testing Framework.

The AI-Aided Hypothesis Generating & Testing Framework was implemented as a modular agent system using the LangChain [23] framework (Figure 1). Following the predefined state graph, the LLM-based agent could execute the workflow and call the tools or LLM models to fulfill the tasks for each step. The agent would use the result context information to reason and adjust its approach as needed until the task was finished.

**Figure 2.**
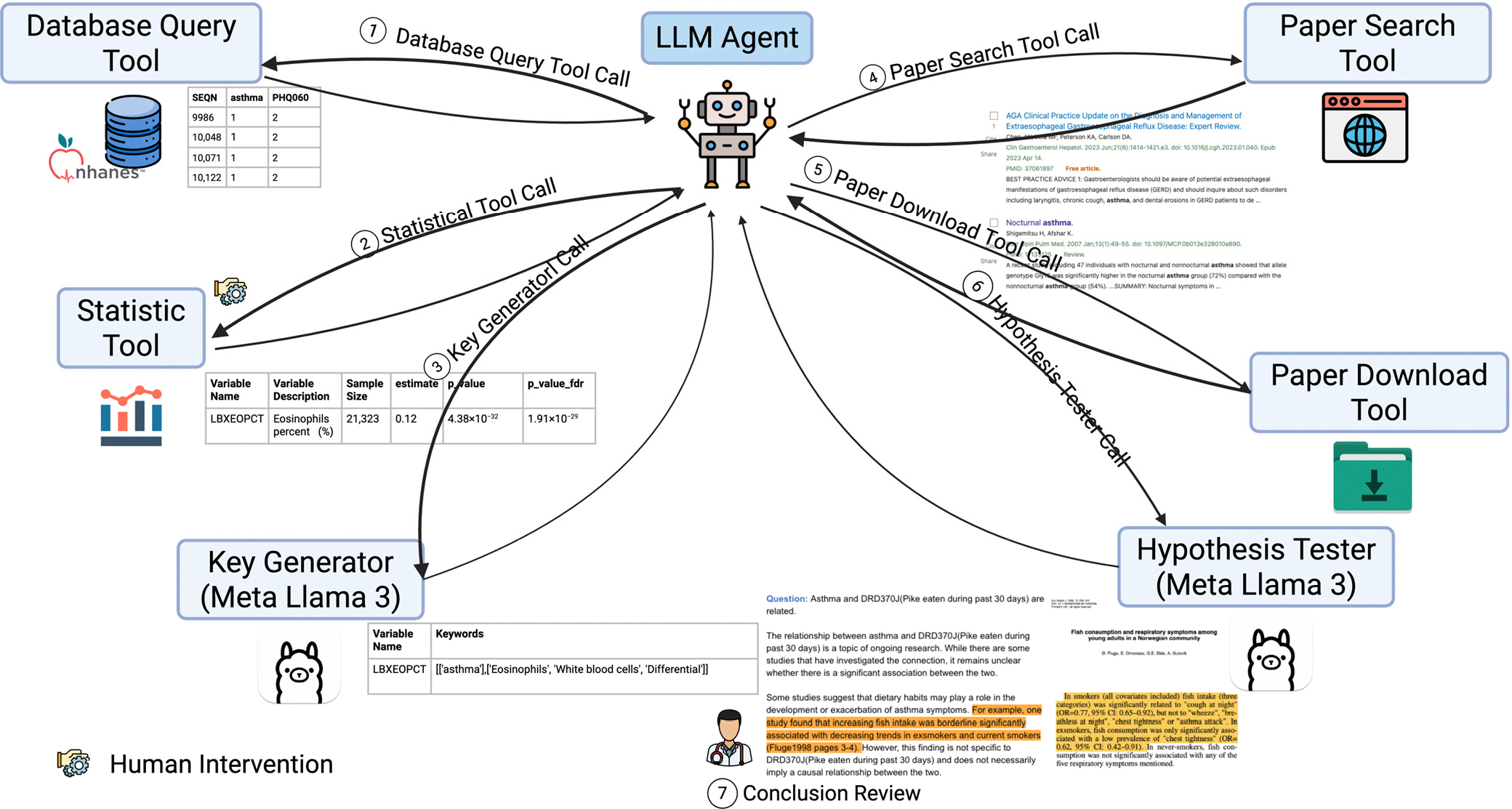
The AI-Aided Hypothesis Generating & Testing Workflow.

The implemented tools included a database query tool, a statistical tool, a paper search tool, and a paper download tool. The LLM models were implemented in the Key Generator and Hypothesis Tester modules. The database query tool could access the NHANES database that was downloaded and stored beforehand. The statistical tool applied multivariable logistic regression to the structured data to get a list of variables significantly associated with current asthma from a statistical viewpoint (Figure 2: ① Database Query Tool Call and ② Statistical Tool Call).

First, for each candidate risk factor, the Database Query Tool was employed to extract data recordings from the NHANES dataset. To ensure the reliability of statistical inferences, we applied variable filtering criteria commonly used in pheWAS analysis [24]. We excluded variables with extreme distributions. In particular, we excluded: binary variables with <20 observations in any group, categorical variables with <20 observations in total across all groups (excluding the dominant group), any variable with <100 total recorded observations, and variables with multiple records per sample (to ensure one-to-one correspondence between observations and samples). Afterward, 3071 candidate variables with valid recordings were retained for multivariable logistic regression analysis, including 274 binary variables, 1208 categorical variables, and 1589 continuous variables.

Next, we applied a series of logistic regression models adjusted for age and gender to examine the associations of each variable with current childhood asthma. Considering the complex multistage survey design of NHANES, we followed the Weighting instruction (https://wwwn.cdc.gov/nchs/nhanes/tutorials/Weighting.aspx, accessed on 14 April 2026) to select and query the corresponding sample weight variable from NHANES for each valid candidate risk factor. To further account for the complex survey design and sampling methods in NHANES—including stratification, clustering, and oversampling of specific subgroups—we constructed a multivariable regression model to incorporate the primary sampling unit variable (SDMVPSU), the stratification variable (SDMVSTRA), and the appropriate sample weight that were provided by NHNAES, ensuring they are representative of the non-institutionalized U.S. population. Significant associations were defined as those with false discovery rate-adjusted P (FDR-P) < 0.01. A total of 141 variables were significant. Human reviews were then conducted to identify and exclude non-informative variables and ensure the stability of regression results (Figure 2: ② Statistical Tool Call). 13 quality control variables were identified by the presence of “comment code” in their descriptions. Four variables were removed due to extreme odds ratio (OR) values, which indicated instability in the model estimates (likely caused by sparse data or quasi-complete separation). An additional 20 variables were removed due to OR = 1, suggesting no association.

Finally, a total of 100 variables, the agent generated tentative affirmative statements regarding their association with current asthma in the following format.

Asthma and Variable Name (Variable Description) are related.

For example, for the variable LBDEONO (peripheral blood eosinophils ≥1000 cells/μL), a claim would be generated as:

Asthma and LBDEONO are related.

Next, the LLM agent would call Key Generator (Figure 2: ③ Key Generator Call) to generate keywords to search in the PubMed database (Prompts in Figure S7).

For example, for the variable SPDBRONC (variable description: Best test FEV1/FVC ratio below Lower Limit of Normal and/or less than 70%; data file description: Spirometry—Pre and Post-Bronchodilator), the generated keywords were: [‘FEV1’, ‘FVC’, ‘Ratio’, ‘Bronchodilator’, ‘Spirometry’].

For DXXGYTOM (variable description: Gynoid total mass; data file description: Dual Energy X-ray Absorptiometry—Android/Gynoid Measurements), the generated keywords are: [‘Mass’, ‘X-ray’, ‘Absorptiometry’, ‘Energy’, ‘Android’, ‘Gynoid’].

For LBXSAL (variable description: Albumin, refrigerated serum (g/dL); data file description: Standard Biochemistry Profile), the generated keywords are: [‘Albumin’, ‘Biochemistry’, ‘Profile’].

To ensure the search was limited to asthma-related publications, the term asthma was combined with all other keywords using the AND operator. This guarantees that every retrieved article is about asthma. Within each group of related concepts, the keywords were combined with the OR operator to capture variations in terminology and maximize coverage. For example, the query term for SPDBRONC (variable description: Best test FEV1/FVC ratio below Lower Limit of Normal and/or less than 70%) was: Asthma AND (FEV1 OR FVC OR Ratio OR Bronchodilator OR Spirometry), Asthma AND (Mass OR X-ray OR Absorptiometry OR Energy OR Android OR Gynoid) for DXXGYTOM (variable description: Dual Energy X-ray Absorptiometry—Android/Gynoid), and Asthma AND (Albumin OR Biochemistry OR Profile) for LBXSAL (variable description: Albumin (g/dL)).

Such keywords were then used as parameters by the Paper Search Tool to search in the PubMed database (Figure 2: ④ Paper Search Tool Call), and the Paper Download Tool, implemented with open-source Python library “paperScraper”, would then download the PDF documents of related scientific papers into a local folder (Figure 2: ⑤ Paper Download Tool Call).

To implement the evidence-based validation step, we integrated the PaperQA library [22] as our Hypothesis Tester module (Figure 2: ⑥ Hypothesis Tester Call). This provided a robust, pre-validated framework for parsing PDFs, retrieving relevant passages via semantic search, and synthesizing summaries with relevance scores. The process, as detailed in [22], involved:

- Text Preparation: The full text of each PDF is parsed into overlapping chunks and embedded to create a searchable vector database.
- Evidence Retrieval & Scoring: For a given claim, the system performs a vector similarity search to retrieve relevant text chunks. Each retrieved chunk was then processed by a summary-dedicated LLM. This LLM was prompted to distill the chunk into a concise evidence summary pertinent to the claim and to assign it a quantitative relevance score (1–10), effectively filtering noise and ranking the most critical evidence.
- Final Verdict Synthesis: The top-ranked evidence summaries were compiled into a final context. A separate LLM then synthesized a final verdict—supporting, rejecting, or expressing uncertainty about the claim—strictly based on this curated body of evidence. This ensured the conclusion was objectively derived from the retrieved literature and provided traceable provenance for each part of the reasoning.

To assess the quality of the LLM-generated keywords, we conducted a survey among domain researchers. The survey presented 20 randomly selected variables and asked researchers to evaluate the corresponding generated keywords across three dimensions on the scale of 1–5 (1 = Strongly Disagree, 5 = Strongly Agree):

- Coverage/Recall: The set captures the major concepts relevant to the variable.
- Precision: The set avoids many clearly off-topic results.
- Redundancy: The set avoids unnecessary duplicates/near-duplicates.

To validate the LLM-generated conclusions, we manually reviewed the variable definitions, LLM-provided summary, and cited evidence (Figure 2: ⑦ Conclusion Review). Variables were classified according to the following criteria:

- Related: published studies explicitly described an association between the variable and asthma (e.g., biomarkers, clinical conditions, or exposures with documented links).
- Indirectly related: no publication explicitly linked the variable to asthma, but the variable reflected a factor that is plausibly connected through intermediate pathways (e.g., physical activity, diet, socioeconomic factors).
- Not related: no evidence from the literature or biological plausibility supporting a connection to asthma.
- Irrelevant: when the LLM could not interpret the variable’s meaning or the data description was insufficient.

To further evaluate the reliability of the manual classifications, we compared our results with those generated independently by three additional large language models (ChatGPT, DeepSeek, and Gemini) (These models were used exclusively in a validation step and were separated from the main workflow which employ only Llama 3 as the LLM in the hypothesis generation or testing process, as illustrated in Figure 1). Each model was provided with the same conclusion generated by the Hypothesis Tester module and asked to classify the variables into the same three categories (directly related, indirectly related, or not related to current asthma). We then compared their outputs with our manual review. Agreement rates were calculated for both a binary grouping (direct + indirect combined as “related” *vs*. “not related”) and the full three-class grouping.

## 4. Results

### 4.1. Screening for Associated Variables

This analysis included 25,839 children with (cases, *n* = 2928) and without (controls, *n* = 22,911) who participated in the 10 NHANES cycles from 2001 to 2020. Multivariable logistic regression was used to test for the association between 10,359 variables in study participants and current asthma. After applying quality control measures, 100 variables were identified as significantly associated with asthma, with a median sample size of 8035 (interquartile range [IQR] = 5168–16,628). Of these 100 variables, 51 were Questionnaire data, 29 were Examination data, 10 were Laboratory data, 9 were Demographics data, and 1 was Dietary data, encompassing all five data types in the NHANES dataset (Figure S1). Table 1 presents the top 10 variables associated with asthma, ranked by FDR-P value in descending order.

**Figure 3.**
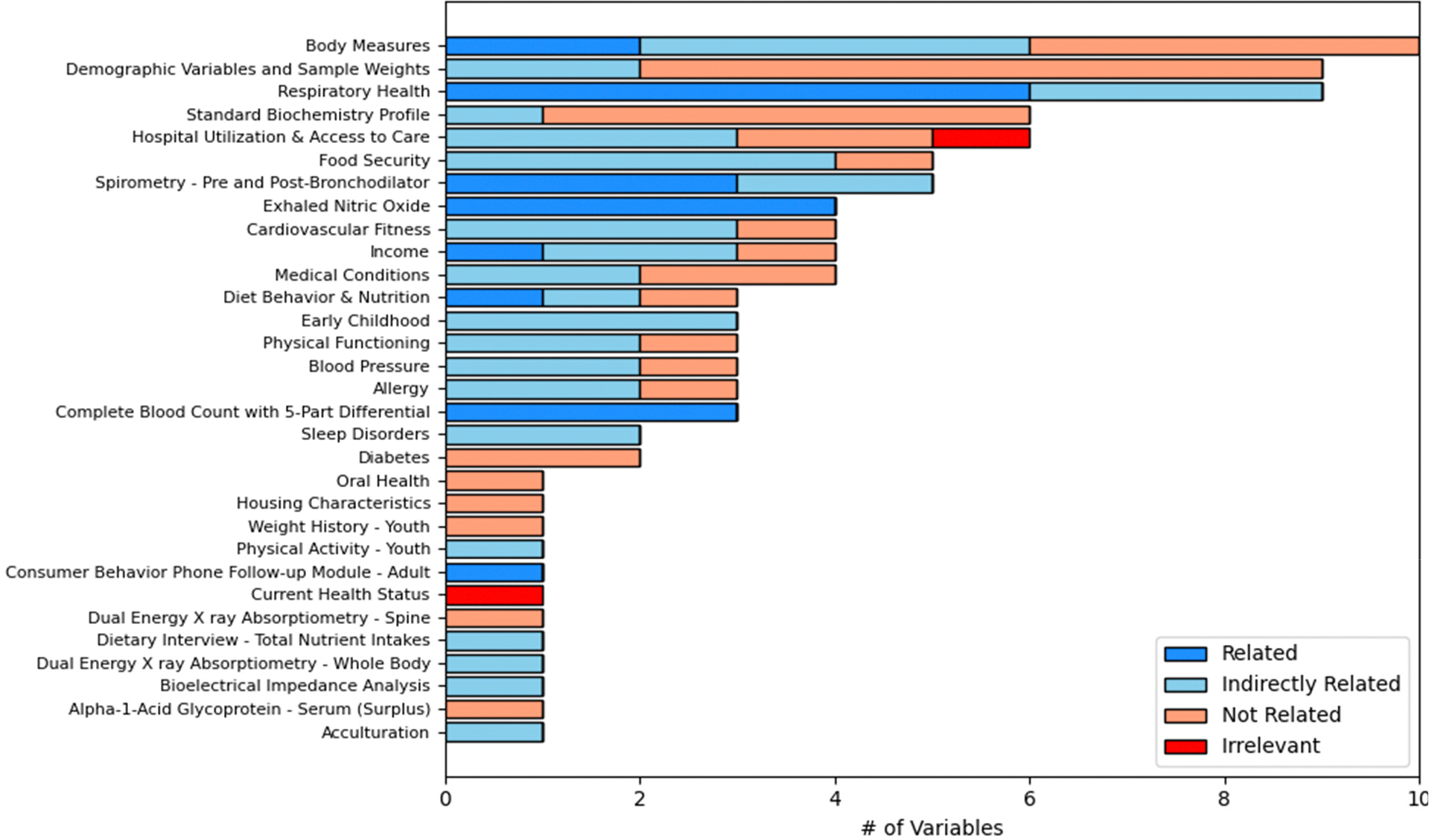
Variable Category Distribution.

Figure 3 shows the category distribution of the selected variables. The top categories with the highest number of variables were “Body Measures”, “Respiratory Health”, “Demographic Variables and Sample Weights”, “Spirometry”, and “Hospital Utilization & Access to Care”. Overall, the variables were well distributed across health conditions, lifestyle and behavioral factors, and laboratory measurements.

### 4.2. Evidence Gathering

After identifying significant variables, the LLM agent generated keywords for a PubMed database search. To evaluate the quality of the LLM-generated keywords, we conducted a survey with 9 domain researchers. Of these 9 researchers, 4 had more than 10 years of research experience in asthma and regularly used PubMed, Google Scholar, or Scopus. The survey presented researchers with 20 randomly selected variables and asked them to assess the corresponding generated keywords along three dimensions: coverage/recall (whether the keywords captured a broad and representative set of concepts), precision (whether the keywords were specific and relevant to the research scope), and redundancy (whether the list avoided unnecessary repetition).

On a 1–5 scale, the keywords received consistently high ratings, with average scores of 4.19 for coverage/recall, 4.15 for precision, and 4.35 for redundancy. These results indicate that the LLM agent produced keywords that were both comprehensive and relevant, with minimal overlap, supporting the reliability of the evidence-gathering process.

With the retrieved list of papers, the Paper Download Tool would try to download the PDF files of all the full-text studies it had access to. However, due to publisher restrictions and paywalls, full-text access could not be guaranteed for all retrieved articles.

Figure S2a shows a histogram of the number of downloaded and loadable studies, which ranged from 15 to 563, with a mean of 320.9 (SD = 106.22) and an interquartile range from 283.25 (25th percentile) to 393.5 (75th percentile).

The LLM’s ability to validate an association was contingent on the evidence that was available in the retrieved studies. As more publications became available, the model was more likely to encounter relevant data and consensus (or conflict) in the literature, leading to a more informed, and thus potentially more reliable, conclusion. Therefore, the sheer volume of retrievable references (measured as the number of loadable papers) served as a crucial factor in gauging the robustness of the LLM’s output.

From Figure S2b, we observe that over 99.0% of the variables had 10 or more full-text studies as references, 95.0% had more than 50, and more than 92.0% had over 100 full-text studies as references. The number of references provided a reasonable basis for the LLM’s conclusions.

### 4.3. Hypothesis Validation

For the 100 variables significantly associated with current asthma, the Hypothesis Tester module called the LLM model to generate answers based on the evidence it gathered. We manually reviewed the generated conclusions and classified 100 variables into three categories (Figure S3):

- 21 variables directly related to current asthma, supported by published studies (Table 2).
- 43 variables with indirect or inferred relationships with current asthma based on the context and background knowledge, though not explicitly mentioned in the available publications (Table 3).
- 34 variables not related to current asthma (Table 4).

For the remaining 2 variables, the LLM failed to understand the exact meaning of the variables or their data file descriptions and provided irrelevant answers (Table S1).

Reproducibility testing with three additional LLMs demonstrated high consistency with our manual classifications. When using the binary grouping of “related” versus “not related”, agreement rates with the manual review were 98% (ChatGPT), 89% (DeepSeek), and 90% (Gemini), indicating strong reproducibility across models. When retaining all three categories, agreement rates were 88%, 81%, and 82%, respectively. These findings show that, similar to human reviewers, LLMs cannot always reach complete agreement, particularly when distinguishing between “Related” and “Indirect Related” relationships, as the boundary between these categories can often be vague.

**Figure 4.**
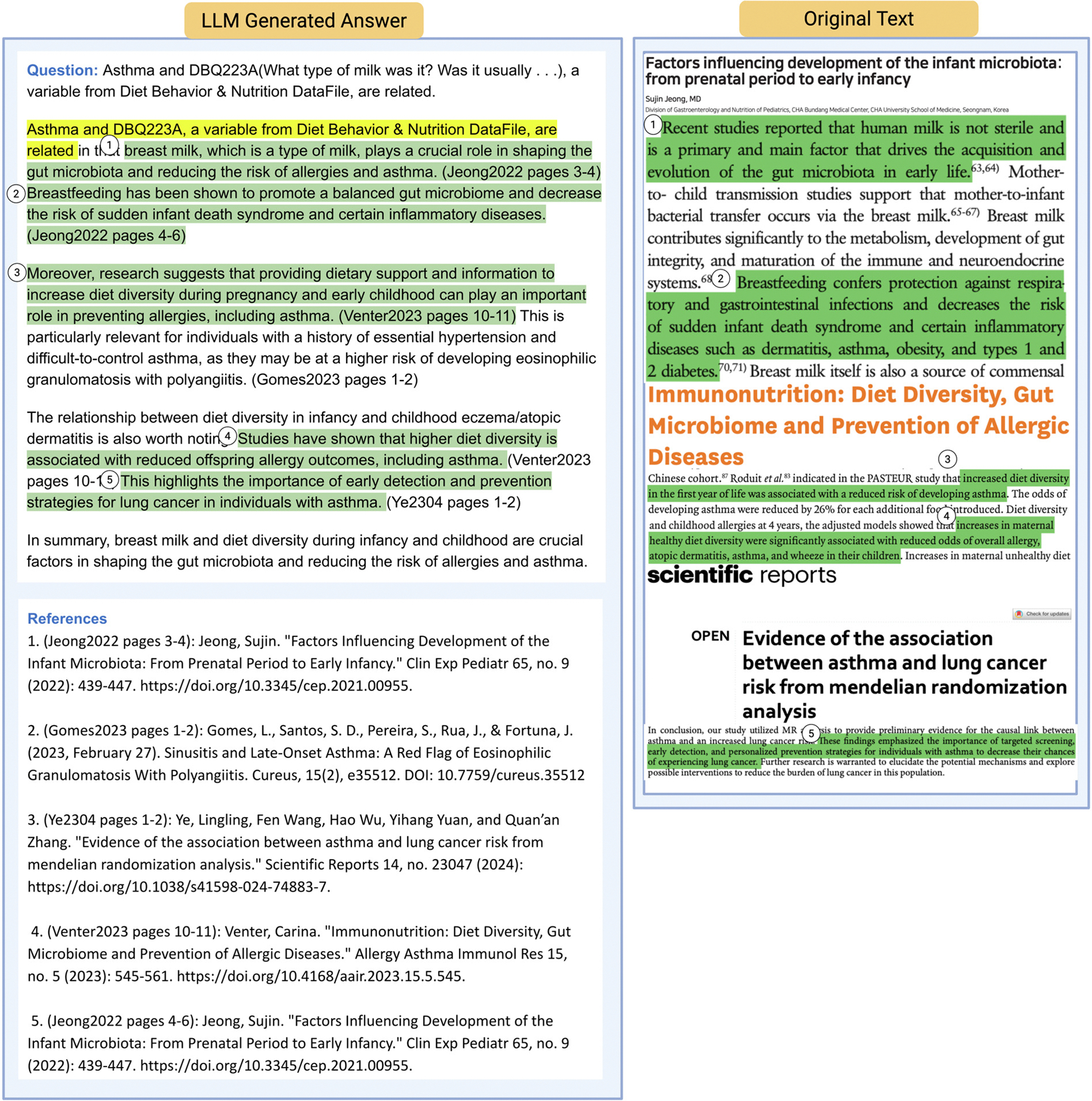
Example of LLM Generated Answer.

Published studies supported the directly related or indirectly related variables. They are positive validations of the workflow, demonstrating that it can reliably recover known associations from observational data. In Figure 4, we show an example using one of the variables identified as related to current asthma, DBQ223A (What type of milk was it? Was it usually ...) and extracted from the Diet Behavior & Nutrition DataFile. The final result was an LLM generated answer to the claim, organized in the format of Question, Answer, and References. In this example, LLM listed 5 references, with each one including a summary of the original text. Figures 3 and S4 illustrate the distribution of variables across the categories found by the LLM to be Not Related, Indirectly Related, or Related to current asthma.

Variables classified as “not related” are those for which the LLM identified no supporting evidence in the provided context linking them to current asthma. This classification reflects the scope and limitations of the information used in the analysis and should not be interpreted as evidence of a definitive absence of association. Rather, it indicates that no documented mechanistic or epidemiological support was found within the analyzed sources. Importantly, some variables with statistically significant associations were classified as “not related” because statistical significance alone does not establish biological or clinical relevance. Such associations may arise from confounding, measurement artifacts, or downstream correlations without substantive supporting evidence. Distinguishing statistical associations from evidence-backed relevance is a central objective of the proposed workflow.

The most notable finding was that, in the absence of documented direct associations in the literature, the LLM systematically identified indirect relationships by integrating contextual information from the provided sources with its embedded domain knowledge. These inferred indirect relationships were not explicitly reported in the reviewed studies, yet they represent structured, evidence-informed connections grounded in existing mechanistic or epidemiological frameworks. As such, they constitute a validated output of the proposed workflow—demonstrating its ability to surface plausible, previously unarticulated links that can guide hypothesis generation and prioritization for subsequent empirical testing.

For the 2 variables with irrelevant answers from LLM (Table S1), a manual inspection of their variable and data file descriptions showed that their descriptions were very similar and lacked discriminative keywords, resulting in irrelevant conclusions. For example, the variable description for HUQ010 is “{First/Next} I have some general questions about {your/SP’s} health. Would you say {your/SP’s} health in general is ...”. This demonstrates that the quality of an LLM’s conclusions heavily relies on the clarity and accuracy of variable descriptions.

## 5. Discussion

This study demonstrates the use of LLM-based models for generating, validating, and rejecting hypotheses through a pilot case study using NHANES data to explore factors potentially associated with childhood asthma. Rather than replacing domain researchers, the proposed workflow restructures their involvement by shifting effort away from exhaustive variable screening and manual literature review toward targeted evaluation and validation. The LLM efficiently synthesizes evidence from the literature to identify variables with documented direct associations and to infer plausible indirect relationships when direct evidence is unavailable. These outputs provide structured, evidence-informed summaries that enable domain experts to focus on critical scientific judgment—assessing biological plausibility, identifying research gaps, and prioritizing variables for follow-up—thereby improving efficiency, transparency, and reproducibility in hypothesis-driven epidemiologic research.

The quality of the answers generated by LLM largely depended on whether the paper search tool module could find and download the most relevant papers on the topic. In this pilot study, we only used PubMed as our search engine, and not all the studies found could be downloaded for full text analysis, due to access limitations. However, >10 references could be downloaded and used as context information for LLM to draw conclusions for 99.0% of the variables of interest.

Searches using keywords composed solely of variable descriptions containing complex chemical compound names (e.g., “5-Hydroxymethyl-2-furancarboxylic”, “N-Nitrosoethylmethylamine (NMEA)”, and “Ethametsulfuron methyl”) often yielded no results in publication databases. Since the Data File Description for chemical compounds included higher-level categories (e.g., “Pesticides”, “Volatile Organic Compounds”, and “Perfluoroalkyl and Polyfluoroalkyl Substances”), we incorporated such categories into the prompts, obtaining markedly improved results, as the LLM successfully generated keywords that returned a substantial number of relevant publications.

While LLM can make logical inferences based on recognized facts, they struggle with vocabulary outside their knowledge base, making them ineffective at generating relevant keywords or answers for variables such as chemical compounds. Although this issue can be mitigated for most variables through proper prompt design, cases ending up with no loadable studies are inevitable. Without reliable evidence to synthesize and validate, the LLM is prone to AI hallucinations.

Understanding concepts and their relationships was valuable not only for generating relevant keywords but also for making reasonable inferences when generating answers or claims. These inferred relationships, combined with expert curation, could help validate generated outputs or inspire new hypotheses.

A relevant example is DRXTCRYP (Beta-cryptoxanthin (mcg)), a variable from the Dietary Interview (Figure S5). Although the LLM did not find direct evidence linking asthma to Beta-cryptoxanthin in the provided context, indirect evidence, such as “some carotenoids have been associated with respiratory morbidity and mortality” and “some studies suggest that certain carotenoids may be related to asthma diagnosis or prevention” was found. Recognizing Beta-cryptoxanthin as a kind of carotenoid, the LLM inferred that DRXTCRYP may be related to asthma.

Another example is LLM inference for MCQ120A (During the past 12 months, {have you/has SP} had ... hay fever?) (Figure S6), a variable from the Medical Conditions database. Although LLM did not find direct evidence for the relationship between asthma and MCQ120A, it found that “atopic dermatitis, which may be related to hay fever, is one of the conditions for which dupilumab (IL-4Rα) is used. This suggests that there may be some overlap between asthma and other allergic conditions like hay fever” and “transmission of asthma susceptibility from mother to offspring through epigenetic changes and glucocorticoid signaling, which highlights the long-term effects of maternal asthma on fetal lung ILC2s and their responsiveness in adulthood. This may imply a link between maternal asthma and respiratory allergies like hay fever” [12]. From these, the LLM inferred that “there may be some overlap between asthma and other allergic conditions like hay fever due to shared mechanisms or susceptibility factors” based on the retrieved literature. This example demonstrates the capacity of LLM to make logical inferences based on underlying disease mechanisms.

Mapping lower-level concepts to higher-level concepts, or vice versa, proved highly effective in identifying relevant references. With refined prompt engineering or LLM accessible tools, general purpose LLMs enhanced with domain specific knowledge of vocabularies and concepts could achieve strong performance in domain-specific tasks.

Retrieval-Augmented Generation (RAG) using reliable publications improves the relevance and contextual grounding of LLM outputs but does not fully eliminate the risk of errors. Current LLM lack the ability to consistently recognize the boundaries of their own knowledge. This contributes to the persistence of hallucinations, underscoring the importance of integrating external validation mechanisms, domain-specific tools, or hybrid frameworks to ensure reliability in epidemiological applications [25].

We acknowledge additional study limitations. First, not all variables in NHANES were measured or available in every participant, leading to variable sample sizes across variables. Second, confounding is possible because we only adjusted our multivariable analyses for age and gender (two variables that influence both exposures and asthma outcomes). However, our goal in this pilot study was to identify associations while avoiding overadjustment. Third, a few variables were named differently across survey years (e.g., annual household income was recorded as “INDHHINC” prior to 2006 and as “INDHHIN2” thereafter). In this study, as the primary objective was to systematically screen a large number of candidate variables and identify those of potential interest, these variables were treated as distinct features rather than being combined, allowing each variable to be evaluated independently for its association with asthma while maintaining a highly efficient, largely automated workflow. Finally, we focused on a single dataset and a single disease (asthma), and thus our findings may not be generalizable to other populations or health outcomes.

In summary, this study introduced a structured, evidence-grounded, and reproducible workflow that integrates large-scale statistical screening with LLM-assisted literature retrieval, synthesis, and validation. While the individual components are not new, their coordinated use enables transparent differentiation between statistically significant associations and biologically supported relevance at scale. Our results highlight the potential use of LLMs in helping conduct efficient clinical research within a standardized, disease-agnostic framework. The proposed workflow can be readily applied to other disease contexts without structural modification, as its core components—statistical screening, evidence retrieval, synthesis, and validation—are not disease specific. Future work will focus on enhancing generalizability and efficiency by integrating domain-specific ontologies, expanding access to diverse databases, and developing specialized modules to better handle complex scientific terms. These advances will further reduce the burden on domain experts while preserving scientific rigor, enabling LLM-assisted workflows to drive innovation across a wide range of biomedical research domains.

## Supplementary Material

Supplementary File

The following supporting information can be found at: https://www.sciepublish.com/article/pii/982, Figure S1: Variable Data Type Distribution. Figure S2: (a) Histogram of number of loadable PDFs; (b) Complementary Cumulative Distribution of loadable PDF number. Figure S3: Different Answer Counts. Figure S4: Variable Category Distribution—Radar Plots. Figure S5: Example of LLM Generated Answer—Logical Inference 1. Figure S6: Key Generator Prompts. Figure S7: Prompts used to classify the LLM generated conclusions. Table S1: Variables with Non Discriminative Descriptions.

## Figures and Tables

**Table 1.** Top 10 Associated Variables.

Variable Name	Variable Description	Estimate	FDR_P	OR	Lower_CI	Upper_CI
DBQ223A	What type of milk was it? Was it usually ...	−0.152	6.97 × 10^−75^	0.859	0.855	0.864
RDQ070	In the past 12 months {have you/has SP} had wheezing or whistling in {your/his/her} chest?	−3.401	6.06 × 10^−59^	0.033	0.028	0.039
PFQ020	{Do you/Does SP} have an impairment or health problem that limits {your/his/her} ability to {walk, run or play} {walk or run}?	−2.175	2.01 × 10^−43^	0.114	0.093	0.138
HUQ010	{First/Next} I have some general questions about {your/SP’s} health. Would you say {your/SP’s} health in general is ...	0.580	1.78 × 10^−42^	1.787	1.687	1.892
LBXEOPCT	Eosinophils percent (%)	0.122	1.91 × 10^−29^	1.130	1.111	1.148
LBDEONO	Eosinophils number (1000 cells/uL)	1.604	2.54 × 10^−26^	4.973	3.947	6.265
RDQ140	[In the past 12 months], {have you/has SP} had a dry cough at night not counting a cough associated with a cold or chest infection lasting 14 days or more?	−1.694	3.29 × 10^−19^	0.184	0.141	0.240
RDQ134	(In the past 12 months), (have you/has SP) taken medication, prescribe by a doctor, for wheezing or whistling?	−2.312	2.63 × 10^−15^	0.099	0.065	0.150
HUQ020	Compared with 12 months ago, would you say {your/SP’s} health is now ...	−0.316	7.02 × 10^−14^	0.729	0.681	0.780
BMXBMI	Body Mass Index (kg/m^2^)	0.045	1.39 × 10^−13^	1.046	1.036	1.056

**Table 2.** Top 10 Related Variables.

Variable Name	Variable Description	Estimate	FDR_P	OR	Lower_CI	Upper_CI
DBQ223A	What type of milk was it? Was it usually ...	−0.152	6.97 × 10^−75^	0.859	0.855	0.864
RDQ070	In the past 12 months {have you/has SP} had wheezing or whistling in {your/his/her} chest?	−3.401	6.06 × 10^−59^	0.033	0.028	0.039
LBXEOPCT	Eosinophils percent (%)	0.122	1.91 × 10^−29^	1.130	1.111	1.148
LBDEONO	Eosinophils number (1000 cells/uL)	1.604	2.54 × 10^−26^	4.973	3.947	6.265
RDQ140	[In the past 12 months], {have you/has SP} had a dry cough at night not counting a cough associated with a cold or chest infection lasting 14 days or more?	−1.694	3.29 × 10^−19^	0.184	0.141	0.240
RDQ134	(In the past 12 months), (have you/has SP) taken medication, prescribe by a doctor, for wheezing or whistling?	−2.312	2.63 × 10^−15^	0.099	0.065	0.150
BMXBMI	Body Mass Index (kg/m^2^)	0.045	1.39 × 10^−13^	1.046	1.036	1.056
ENXTR2Q	Trial 2 measurement of fractional concentration of orally exhaled nitric oxide (FENO) in parts per billion	0.019	9.58 × 10^−9^	1.020	1.015	1.024
ENXMEAN	Mean of two reproducible FENO measurements, in parts per billion	0.019	1.28 × 10^−8^	1.020	1.015	1.025
ENXTR1Q	Trial 1 measurement of fractional concentration of orally exhaled nitric oxide (FENO) in parts per billion	0.020	2.77 × 10^−8^	1.020	1.015	1.026

**Table 3.** Indirectly Related Variables.

Variable Name	Variable Description	Estimate	FDR P Value	Odds Ratio	Lower_95 % CI	Upper_95 % CI
PFQ020	{Do you/Does SP} have an impairment or health problem that limits {your/his/her} ability to {walk, run or play} {walk or run}?	−2.175	2.01 × 10^−43^	0.114	0.093	0.138
MCQ080E	Has a doctor or health professional ever told you that {SP} was overweight?	−0.641	9.59 × 10^−11^	0.527	0.448	0.619
BMXWT	Weight (kg)	0.012	1.69 × 10^−9^	1.013	1.009	1.016
PFQ041	Does {SP} receive Special Education or Early Intervention Services?	−0.561	5.21 × 10^−9^	0.571	0.488	0.667
ACD040	Now I’m going to ask you about language use. What language(s) {do you/does SP} usually speak at home? {Do you/Does he/Does she} speak only Spanish, more Spanish than English, both equally, more English than Spanish, or only English?	−0.291	1.48 × 10^−8^	1.338	1.229	1.457
HUQ090	During the past 12 months, that is since {DISPLAY CURRENT MONTH} of {DISPLAY LAST YEAR}, {have you/has SP} seen or talked to a mental health professional such as a psychologist, psychiatrist, psychiatric nurse or clinical social worker about {your/his/her} health?	−0.514	6.70 × 10^−8^	0.598	0.511	0.701
RDD120	[In the past 12 months], how many times {have you/has SP} gone to the doctor’s office or the hospital emergency room for one or more of these attacks of wheezing or whistling?	0.346	7.34 × 10^−8^	1.413	1.276	1.565
HUQ071	{During the past 12 months, were you/{was} SP} a patient in a hospital overnight? Do not include an overnight stay in the emergency room.	0.875	7.34 × 10^−8^	0.417	0.319	0.545
CVDEXSTS	CV fitness exam status	−0.541	9.74 × 10^−8^	1.719	1.513	1.951
BPACSZ	Cuff size (cm) (width × length)	0.298	3.36 × 10^−7^	1.347	1.224	1.483

**Table 4.** Top 10 Unrelated Variables.

Variable Name	Variable Description	Estimate	FDR_P	OR	Lower_CI	Upper_CI
HUQ020	Compared with 12 months ago, would you say {your/SP’s} health is now ...	−0.316	7.02 × 10^−14^	0.729	0.681	0.780
BMXARMC	Arm Circumference (cm)	0.049	1.34 × 10^−10^	1.050	1.037	1.063
BMXWAIST	Waist Circumference (cm)	0.015	3.60 × 10^−10^	1.015	1.011	1.020
SIALANG	Language of the Sample Person Interview Instrument	−0.666	9.76 × 10^−10^	0.514	0.429	0.615
FIALANG	Language of the Family Interview Instrument	−0.678	4.95 × 10^−9^	0.508	0.419	0.615
DMDCITZN	{Are you/Is SP} a citizen of the United States? [Information about citizenship is being collected by the U.S. Public Health Service to perform health related research. Providing this information is voluntary and is collected under the authority of the Public Health Service Act. There will be no effect on pending immigration or citizenship petitions.]	−1.127	1.19 × 10^−7^	0.324	0.228	0.460
CVDEXCL4	Excluded from exam due to selected asthma symptoms	−3.469	8.70 × 10^−7^	0.031	0.012	0.078
RIDRETH1	Recode of reported race and Hispanic origin information	−0.316	5.06 × 10^−6^	1.118	1.074	1.164
OHQ576Q	How old in months or years was {SP} when {he/she} stopped taking prescription fluoride drops or fluoride tablets?	0.001	7.49 × 10^−6^	1.001	1.000	1.001
HUQ051	{During the past 12 months, how/How} many times {have you/has SP} seen a doctor or other health care professional about {your/his/her} health at a doctor’s office, a clinic or some other place? Do not include times {you were/s/he was} hospitalized overnight, visits to hospital emergency rooms, home visits or telephone calls.	0.022	9.70 × 10^−6^	1.02	1.014	1.03

## Data Availability

The data used in this study are publicly available from the National Health and Nutrition Examination Survey (NHANES). These datasets can be accessed free of charge through the official website of the Centers for Disease Control and Prevention (CDC) at https://www.cdc.gov/nchs/nhanes/ (accessed on 14 April 2026). No additional restrictions apply to the use of these data.
