# Supplementary material for: Harnessing Artificial Intelligence for Hypothesis Generation in Childhood Asthma: Insights from NHANES
Source: J Respir Biol Transl Med. Author manuscript; Available in PMC 2026 Jun 19. (PMC13278482; doi:10.70322/jrbtm.2026.10003)
Supplement: Supplementary File [file NIHMS2180431-supplement-Supplementary_File.pdf]

Table S1. Variables with Non Discriminative Descriptions.

Variable Name	Variable Description	Data File Description
HUQ010	{First/Next} I have some general questions about {your/SP's} health. Would you say {your/SP's} health in general is . . .	Hospital Utilization & Access to Care
HSD010	Next I have some general questions about {your/SP's} health. Would you say {your/SP's} health in general is . . .	Current Health Status

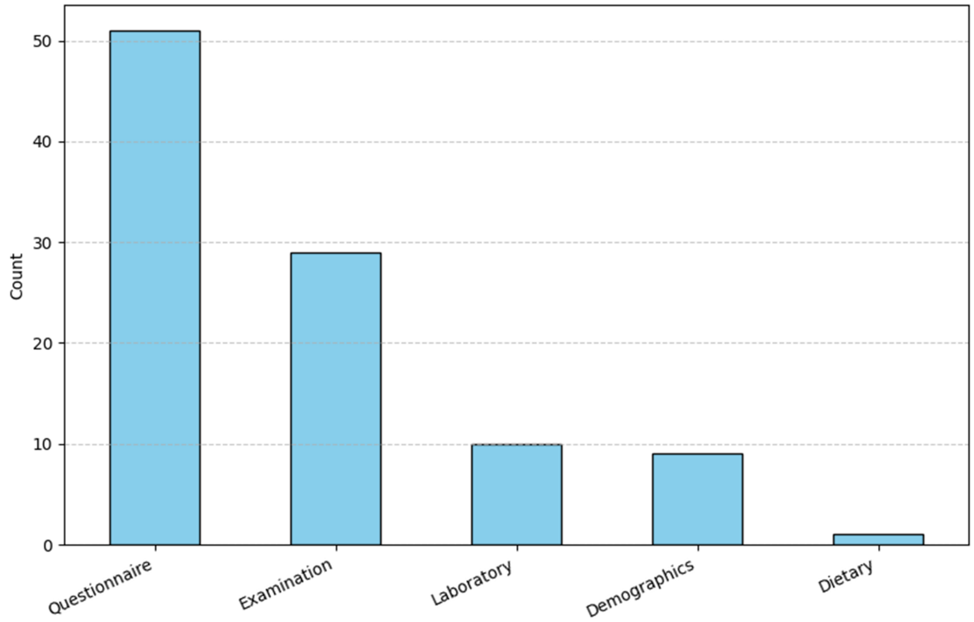

Figure S1. Variable Data Type Distribution.

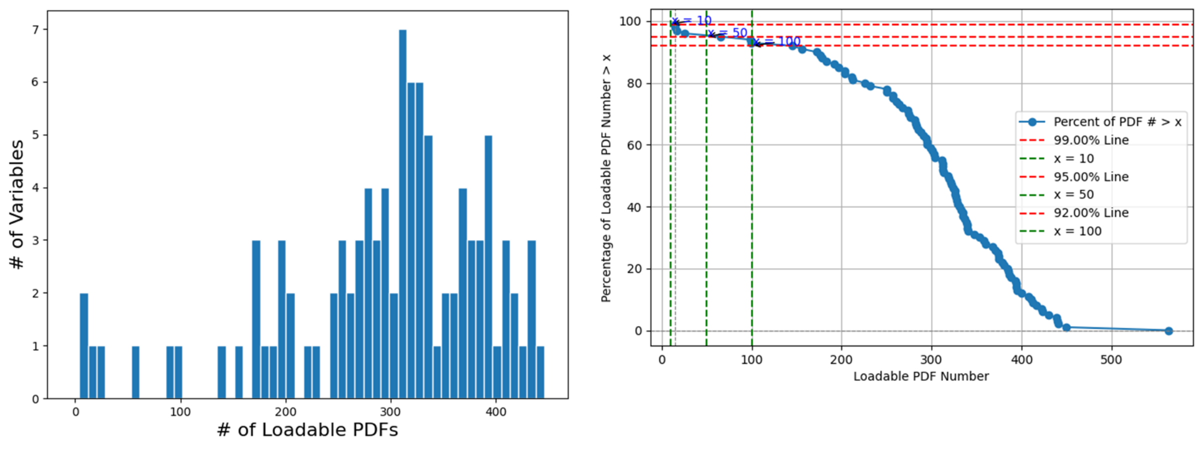

(**a**) (**b**)

Figure S2. (**a**) Histogram of number of loadable PDFs; (**b**) Complementary Cumulative Distribution of loadable PDF number.

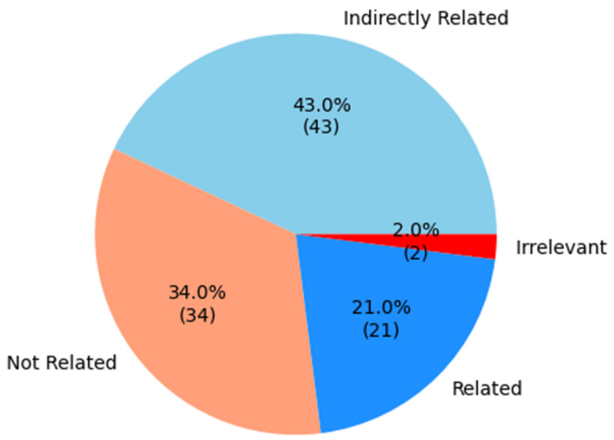

Figure S3. Different Answer Counts.

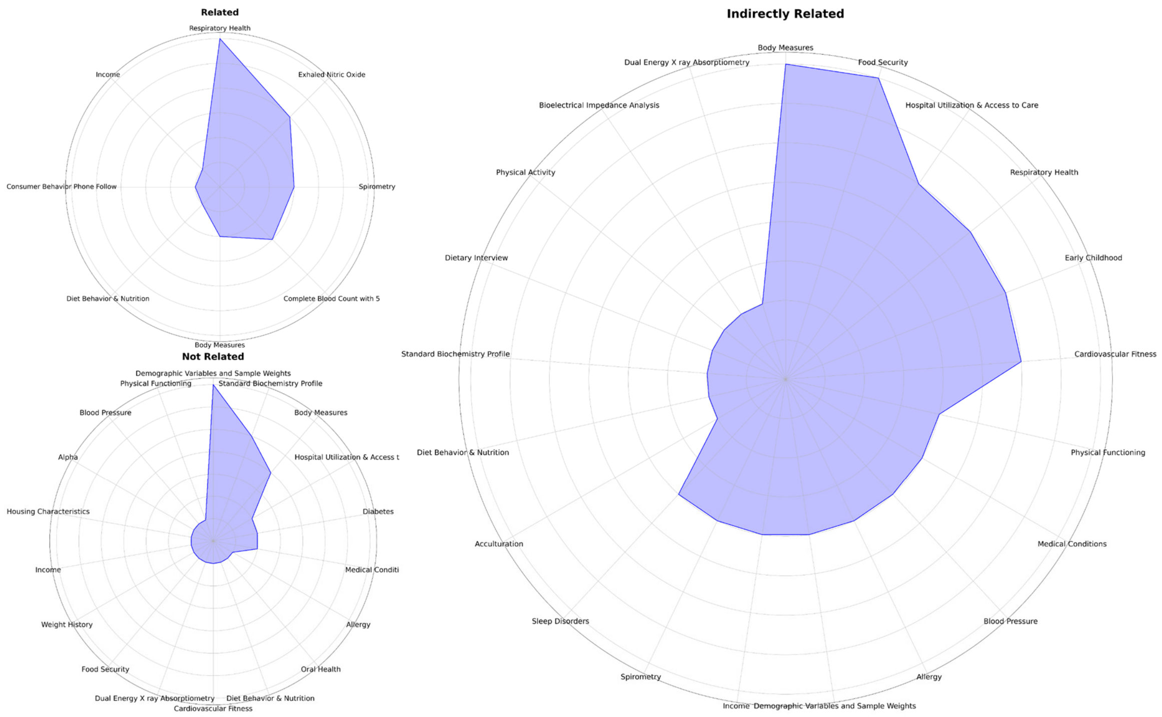

Figure S4. Variable Category Distribution—Radar Plots.

Question: Asthma and DRXTCRYP(Beta-cryptoxanthin (mcg)), a variable from Dietary Interview - Total Nutrient Intakes DataFile, are related.

Asthma and DRXTCRYP (Beta-cryptoxanthin) may be related, as suggested by some studies. While there is no direct evidence linking asthma to Beta-cryptoxanthin in the provided context, some carotenoids have been associated with respiratory morbidity and mortality (Yang et al., 2023).

The relationship between carotenoids and asthma is not well-established, but some studies suggest that certain carotenoids may be related to asthma diagnosis or prevention (Kohandel Gargari et al., 2025). For example, feature interpretation using SHapley Additive exPlanations (SHAP) values identified significant predictors for asthma diagnosis, including dietary fat intake (Kohandel Gargari et al., 2025).

However, there is no specific information about the relationship between asthma and Beta-cryptoxanthin in the provided context. Further research would be necessary to determine if Beta-cryptoxanthin has a direct impact on asthma.

Figure S5. Example of LLM Generated Answer—Logical Inference 1.

Key Generator Prompt:

You are an expert language model tasked with summarizing data and extracting keywords from variable descriptions.

Choose only 2 to 5 important keywords from the following variable description: "{variable_description}" and data file description:"{datafile_description}"

Use only key nouns and significant terms. Do not include general terms or the word 'asthma'. No duplicates.

Figure S6. Key Generator Prompts.

ChatGPT/DeepSeek/Gemini Prompt:

Read the following passage and decide whether Asthma is related/not related/indirectly related to the variable of interest.

Figure S7. Prompts used to classify the LLM generated conclusions.
